# Impact of physical activity, sedentary behaviour and muscle strength on bone stiffness in 2–10-year-old children-cross-sectional results from the IDEFICS study

**DOI:** 10.1186/s12966-015-0273-6

**Published:** 2015-09-17

**Authors:** Diana Herrmann, Christoph Buck, Isabelle Sioen, Yiannis Kouride, Staffan Marild, Dénes Molnár, Theodora Mouratidou, Yannis Pitsiladis, Paola Russo, Toomas Veidebaum, Wolfgang Ahrens, on behalf of the IDEFICS consortium

**Affiliations:** Department of Epidemiological Methods and Etiological Research, Leibniz Institute for Prevention Research and Epidemiology - BIPS, Achterstr. 30, 28359 Bremen, Germany; Department of Biometry and Data Management, Leibniz Institute for Prevention Research and Epidemiology - BIPS, Achterstr. 30, 28359 Bremen, Germany; Department of Public Health, Ghent University, 4K3, De Pintelaan 185, 9000 Ghent, Belgium; Research and Education Institute of Child Health, 138 Limassol Ave, #205, 2015 Strovolos, Cyprus; Department of Paediatrics, Queen Silvia Children’s Hospital, University of Gothenburg, Rondvägen 15, 41685 Gothenburg, Sweden; Department of Pediatrics, Medical Faculty, University of Pecs, Jozsef A. u. 7, 7623 Pecs, Hungary; GENUD (Growth, Exercise, Nutrition and Development) Research Group, University of Zaragoza, C/Domingo Miral s/n, 50009 Zaragoza, Spain; Centre for Sport and Exercise Science and Medicine (SESAME), University of Brighton, Welkin House, 30 Carlisle Road, Eastbourne, BN20 7SN UK; Institute of Food Sciences, National Research Council, Via Roma 64, 83100 Avellino, Italy; Department of Chronic Diseases, Centre of Behavioural and Health Sciences, National Institute for Health Development, Hiiu 42, 11619 Tallinn, Estonia; Faculty of Mathematics and Computer Science, Bremen University, Bibliothekstraße 1, 28359 Bremen, Germany

**Keywords:** Bone stiffness, Physical activity, Sedentary behaviour, Accelerometer, Quantitative ultrasound, Quantitative evidence, Weight-bearing exercise, Muscle strength

## Abstract

**Background:**

Physical activity (PA), weight-bearing exercises (WBE) and muscle strength contribute to skeletal development, while sedentary behaviour (SB) adversely affects bone health. Previous studies examined the isolated effect of PA, SB or muscle strength on bone health, which was usually assessed by x-ray methods, in children. Little is known about the combined effects of these factors on bone stiffness (SI) assessed by quantitative ultrasound. We investigated the joint association of PA, SB and muscle strength on SI in children.

**Methods:**

In 1512 preschool (2- < 6 years) and 2953 school children (6–10 years), data on calcaneal SI as well as on accelerometer-based sedentary time (SED), light (LPA), moderate (MPA) and vigorous PA (VPA) were available. Parents reported sports (WBE versus no WBE), leisure time PA and screen time of their children. Jumping distance and handgrip strength served as indicators for muscle strength. The association of PA, SB and muscle strength with SI was estimated by multivariate linear regression, stratified by age group. Models were adjusted for age, sex, country, fat-free mass, daylight duration, consumption of dairy products and PA, or respectively SB.

**Results:**

Mean SI was similar in preschool (79.5 ± 15.0) and school children (81.3 ± 12.1). In both age groups, an additional 10 min/day in MPA or VPA increased the SI on average by 1 or 2 %, respectively (*p* ≤ .05). The negative association of SED with SI decreased after controlling for MVPA. LPA was not associated with SI. Furthermore, participation in WBE led to a 3 and 2 % higher SI in preschool (*p* = 0.003) and school children (*p* < .001), respectively. Although muscle strength significantly contributed to SI, it did not affect the associations of PA with SI. In contrast to objectively assessed PA, reported leisure time PA and screen time showed no remarkable association with SI.

**Conclusion:**

This study suggests that already an additional 10 min/day of MPA or VPA or the participation in WBE may result in a relevant increase in SI in children, taking muscle strength and SB into account. Our results support the importance of assessing accelerometer-based PA in large-scale studies. This may be important when deriving dose–response relationships between PA and bone health in children.

## Background

High levels of physical activity (PA) have been found to optimize skeletal development early in life, thus preventing age-related bone loss and osteoporotic fractures [1–4]. The positive impact of moderate (MPA), vigorous (VPA) or moderate-to-vigorous PA (MVPA) on bone health in children has been demonstrated in several observational studies [5–11]. In school-based interventions an osteogenic effect of WBE such as jumping or ballgames has been observed. The effect of high-impact PA has been largely explained by the muscle force and strength acting on bone [2, 12–18]. Thus, muscle strength and muscle mass play an important role in bone development during growth [19].

International PA guidelines for children from the World Health Organization (WHO) recommend one hour of MVPA per day, including VPA or bone-strengthening exercises on at least three days a week [20]. However, a large number of studies have indicated that most children spend insufficient time in MVPA [6, 7, 21–23]. The time previously spent in MVPA may be replaced by the increasing time children spend in sedentary behaviours such as watching television or playing computer games that may adversely affect bone health [5, 24, 25]. According to previous studies, the adverse effect of sedentary behaviours on bone health may be counteracted by additional high-impact PA [26, 27].

The variety of methods for assessing and operationalizing PA and bone health hamper the comparison of studies, particularly for investigating consistent dose–response relationships and bone-related PA recommendations. This is further complicated by the fact that usually only the isolated osteogenic effect of either habitual PA, different types of WBE or sedentary behaviours has been examined. The osteogenic effect of different PA intensities combined with sedentary behaviour in children is poorly investigated. In particular, there is a lack of quantitative evidence on the association of PA and WBE with bone health in children younger than five years [6].

In the IDEFICS study (Identification and prevention of dietary- and lifestyle- induced health effects in children and infants), a large European sample of children aged 2–10, bone stiffness index (SI), as an indicator for bone health, was measured using quantitative ultrasound (QUS) [28]. We comprehensively assessed habitual PA levels, sedentary behaviour and physical fitness, which made it possible for us to simultaneously investigate the association of these lifestyle factors with SI in children. In detail, we examined the effect of objectively measured average PA levels, SED, LPA, MPVA, VPA and MVPA as well as of parental-reported leisure time PA, WBE and screen time on SI in preschool (2- < 6 years) and school children (6–10 years). We additionally investigated the association of muscular fitness and fat-free mass (FFM) on SI separately as well as in combination with PA and sedentary behaviour. Both, muscular fitness and FFM have been used as indicators for muscle strength and muscle mass in previous studies [8, 29–31].

## Methods

### Study sample

The IDEFICS study, a prospective population-based cohort study, examined more than 18,000 2–11-year-old children, from eight European countries (Sweden, Germany, Hungary, Italy, Cyprus, Spain, Belgium and Estonia) to investigate associations of biological and behavioural factors on lifestyle diseases. The study was conducted according to the standards of the Declaration of Helsinki. All participating centres obtained ethical approval from their responsible authority. Participating children and their parents provided oral and written informed consent for each examination and for the storage of personal data. Children and their parents were allowed to opt out of single examination modules, e.g. blood collection, accelerometry or QUS measurements. The study design and examinations done have been described in detail elsewhere [32, 33]. Our sample is based on 16,228 children examined at baseline (T0, 2007/08), and 2555 newly recruited children examined at follow-up (T1, 2009/10).

**Fig. 1:**
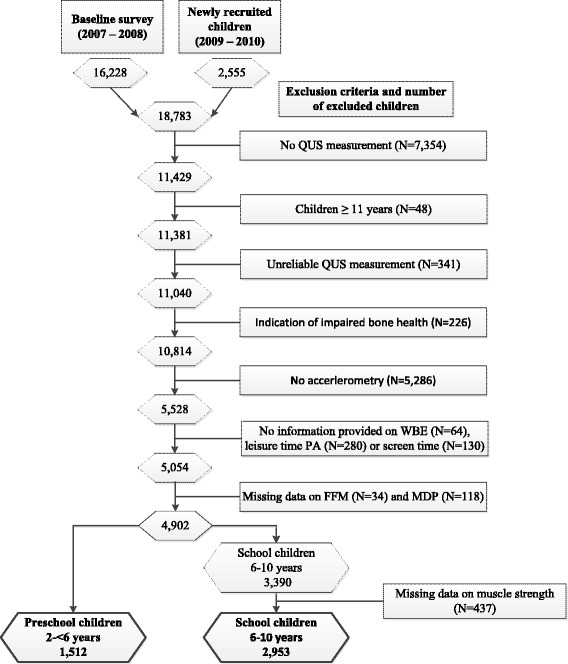
Number of included and excluded children per exclusion criteria for the analysis group

The exclusion criteria and the number of children of the final analysis are summarized in Fig. 1. We considered children 2–10 years of age with their first QUS measurement at T0 (*N* = 7539) or T1 (*N* = 3842). We excluded children with invalid QUS measurements (*N* = 341) and with an indication of impaired bone health, i.e. with diseases or receiving medical treatments affecting the bone (*N* = 226). Further, we excluded children without accelerometer measurements (*N* = 5286), no parental report on participation in a sports club and no information on WBE (*N* = 64), leisure time PA (*N* = 280) or screen time (*N* = 130). Children were also excluded, if data on FFM (*N* = 34) and consumption of milk and dairy products (MDP) (*N* = 118) were missing. In school children, we considered only children who participated in fitness tests including jumping distance and handgrip strength. These restrictions left 1512 preschool and 2953 school children for analysis.

### Bone stiffness

SI was measured on the left and right calcaneus using QUS (Achilles Lunar Insight^TM^ GE Healthcare, Milwaukee, WI, USA) and was based on two parameters, broadband ultrasound attenuation (dB/MHz) and speed of sound (m/s) [34]. QUS measurements are correlated with DXA measurements and are used as a valid tool for indicating the risk of osteoporotic fractures [35, 36]. In children, however, the clinical usefulness of QUS has not yet been investigated, and comparison studies showed inconsistent correlations with DXA [34, 37]. The detailed method and application of QUS is described elsewhere [28, 38, 39]. To examine the reliability of Achilles devices, 60 children were repeatedly measured on either the right (*N* = 30) or left (*N* = 30) calcaneus. The precision of Achilles measurements was calculated using the percent root-mean-square coefficient of variation (CV_RMS_) in accordance with the Conference of Radiation Control Program Directors (CRCPD) Task Force on Bone Densitometry [40]. CV_RMS_ for SI was 9.2 % on the right foot and 7.2 % on the left foot. Furthermore, in our sample, the absolute difference of the left and right SI measurement was on average 10.3 units (standard deviation 12.5 units) and ranged from 0 to 112 units. A small reliability study that was conducted in a convenience sample (*N* = 91) based on five different Achilles devices used in the IDEFICS study confirmed the significant discrepancy of the SI measurements between the left and right foot (multilevel regression analysis: β = 0.45, *p* = 0.05, unpublished data). To control for this discrepancy we set a limit for the absolute SI difference between SI of the left and right calcaneus and excluded 3 % of the QUS sample having the highest SI difference (97^th^ SI percentile: 45 units SI difference). We calculated the mean SI of both feet as a proxy for bone status of the lower limbs and hip.

### Physical activity and sedentary behaviour

In the IDEFICS study, PA was objectively measured using Actigraph uniaxial accelerometers (ActiTrainer or GT1M; Actigraph, LLC, Pensacola, FL, USA) in a subgroup of about 9000 children. Both types of accelerometers have been observed to measure comparable MVPA levels. However, the authors noted the lower comparability for lower PA levels, thus results should be interpreted carefully [41]. Because the number of accelerometer was limited in the IDEFICS study, accelerometers could not be offered to all children. We had no selection criteria for the distribution of an accelerometer. If an accelerometer was available and could be offered to the child, the child was happy to wear it. Sociodemographic variables did not vary between children who wore an accelerometer and children who did not. Thus, we assume that the distribution of accelerometers was randomized and the results are not systematically distorted. Children had to wear the device on their right hip and had to take it off during water-based activities and bedtime. Data were considered valid when the child wore the accelerometer for three consecutive days, including one weekend day, for at least six hours per day. In our analysis, we used accelerometer data with 60 s (s) epoch. Non-wearing time was defined as 20 min or more of consecutive zero counts [21]. The average PA levels of the children were defined by counts per minute (cpm). Intensity levels were classified as sedentary time (SED, ≤100 cpm), LPA (>100- < 2296 cpm), MPA (≥2296- < 4012 cpm), VPA (≥4012 cpm) and MVPA (≥2296 cpm) based on the cut-off values proposed by Evenson that are published by Trost et al., 2011 [42]. For each intensity level the cumulative duration was calculated in minutes per day. The total valid wearing time of the device was assessed and expressed as average hours per day.

In addition to the objectively measured PA, the child’s parental-reported PA and sedentary behaviour were considered in our analysis. Therefore, children’s WBE, leisure time PA and screen time were reported by parents using a questionnaire. The variable WBE was based on the two questions 1) “Is your child a member of a sports club?” (response options: yes / no) and 2) “What kind of sport does your child do in a sports club?” If a child participated in sport club activities, four types of sport typical for each respective country and an open category to record other sports that were not listed were offered as response options. All reported types of sport were classified according to their loading and categorised into: (i) moderate or high mechanical loads on the lower limbs (ballgames, gymnastics, dancing, skating, martial arts, and athletics), and (ii) no or low mechanical loads (swimming, biking and horseback riding). The latter also included children for whose parents answered that they did not participate in sports club activities in question 1 (no sports).

The variable leisure time PA was based on the “Outdoor Playtime Recall Questions” [43]. Parents were asked how many hours (h) and minutes (m) their child spent playing outdoors on a typical weekday (weekd_h, weekd_m) and weekend day (weeken_h, weeken_m) the previous month. In addition, parents were asked how many hours (club_h) and minutes (club_m) per week the child spent doing sport in a sports club. Finally, the variable leisure time PA was calculated as 5*(weekd_h +weekd_m/60) + 2*(weeken_h + weeken_m/60)+ (club_h+ club_min/60) and expressed as hours per week.

Sedentary behaviours, such as watching TV or playing computer games were used as a proxy for reported sedentary time [27]. Parents were asked to recall the usual duration their child watched (i) TV, videos, and DVDs, and (ii) the duration their child used the computer and game console on a normal weekday and weekend day. For both questions, six response categories were offered and converted into the following scoring system: not at all =0, <30 min =1, <1 h =2, 1- < 2 h =3, 2-3 h =4, and >3 h =5. Screen time was calculated separately for weekdays (weekd_score) and weekend days (weeken_score) by adding up the converted responses of questions (i) and (ii). The total screen time in hours per week was calculated as [(weekd_score*5) + (weeken_score*2)].

### Assessment of muscle strength

Physical fitness tests were conducted only among school children and adapted from the ALPHA (Assessing Levels of Physical Activity) and FITNESSGRAM test battery [29, 44, 45]. Jumping distance and handgrip strength were considered to indicate muscle strength in the lower and upper limbs, respectively [29]. Jumping distance was assessed using a standing broad jump test, measured to the nearest 1.0 cm. Handgrip strength was measured to the nearest 0.1 kg using a digital handgrip dynamometer (Takei TKK 5401/5101). Each child had two attempts per test and the maximum value of both attempts was considered for our analysis.

### Assessment of fat-free mass

FFM (kg) was used as a proxy for skeletal muscle mass, which has been reported to be positively associated with bone strength [30, 31]. It was calculated based on height (Stadiometer SECA 225), weight, and leg-to-leg bioelectrical impedance (both measured with Tanita scale BC420 MA) using the Tyrrell formula [46]. As height and weight are strongly correlated with FFM, we did not consider either of them as an adjustment variable. In the current analysis, on the one hand, the association of FFM with SI was examined without considering PA behaviour. On the other hand, the association between PA behaviour and SI was additionally controlled for FFM, which is in accordance with previous studies [7–9].

### Assessment of co-variables

The consumption of milk and dairy products (MDP) was considered as an indicator for calcium intake and estimated based on the habitual consumption frequency of milk, yoghurt, and cheese as reported by parents using a food frequency questionnaire that was developed in the IDEFICS study [47, 48]. The response categories ranged from ‘Never/less than once a week’ to ‘Four or more times per day’ and were converted into the weekly frequency of MDP consumption.

Exposure to sunlight is the most important source for vitamin D synthesis that contributes to bone mineralization [49]. Hence, we adjusted for mean daylight duration (±0.1 h) which was calculated for each examination month in each location, using astronomical tables [50].

### Statistical analyses

The associations of PA, sedentary behaviour and muscle strength with SI were analysed using multivariate linear regression models. Data were checked for normality and linearity using residual plots. Regression analyses were conducted for each variable of PA behaviour (LPA, MPA VPA, MVPA, average PA level, WBE, leisure time PA), sedentary behaviour (SED, screen time) and muscle strength (FFM, jumping distance, handgrip strength). Objectively measured and reported variables were analysed and presented separately. In a first model, we adjusted for age, sex and country (Model 1). The second model was additionally adjusted for FFM (unless FFM was an independent variable), MDP and daylight duration (Model 2). A third model was conducted to additionally adjust model 2 for the time of either accelerometer-based SED or MVPA, or respective reported PA/ sedentary behaviours (Model 3). Thus, except for the average PA level, each independent variable was additionally adjusted as follows: the *PA intensities LPA, MPA, VPA and MVPA* were each adjusted for SED; *SED* was adjusted for MVPA; *variables for muscle strength* for average PA level; *screen time* for reported leisure time PA; and *leisure time PA and WBE* for reported screen time. In school children, we additionally adjusted model 3 for jumping distance and handgrip strength (Model 4). Except for the model for average PA level, all models that included accelerometer data were adjusted for valid wearing time of the accelerometer. To allow better interpretation of the regression coefficients, accelerometer-based variables were converted as follows: average PA level as 100 cpm, SED and LPA as hours/day and MPA, VPA and MVPA as 10 min/day.

Analyses were conducted for boys and girls together, since no moderating effect of sex on the association between PA and SI was detected. Regression models were stratified for preschool (2- < 6 years) and school children (6–10 years). This was decided due to the lack of evidence in children younger than six years, and the lack of fitness data in preschool children. Furthermore, previously published age-, sex-, and height-specific SI percentile values based on the IDEFICS sample indicated a negative age-trend of SI in preschool children and a positive age-trend in school children [28]. The apparent decline of SI in preschool age may be explained by the increased growth velocity in early childhood, where bone turnover processes may not have fully compensated for growth [51].

Based on the described models, we performed sensitivity analyses with accelerometer data using 15 s epochs that were not available for all children (*N* = 3519).

Level of significance was set at α = 0.05. Analyses were performed using SAS 9.3 (SAS Institute Inc., Cary, NC, USA).

## Results

### Study sample characteristics

The characteristics of the study sample are shown in Table 1. Differences in mean SI by age and sex were negligible. Preschool and school children wore the accelerometer on average 11.5 and 12.2 h per day, respectively. The accelerometer-based average PA levels were slightly higher in preschool compared to school children. The mean time spent in LPA, MPA and VPA as well as the reported leisure time PA were similar for both age groups. School children were more engaged in WBE, but also reached a higher mean of SED and reported screen time compared to preschool children. In both age groups, boys had slightly higher mean values in all PA variables, except LPA and SED, as well as a slightly higher reported screen time. Finally, compared to school girls, school boys had slightly higher mean values in the fitness tests.

**Table 1:** Characteristics of preschool (2- < 6 years) and school (6–10 years) children, stratified by sex

	Preschool children (2- < 6 years)			Primary school children (6–10 years)		
	Boys	Girls	All	Boys	Girls	All
	Mean ± SD	Mean ± SD	Mean ± SD	Mean ± SD	Mean ± SD	Mean ± SD
Number	804	708	1512	1409	1544	2953
Age (years)	4.4 ± 0.9	4.5 ± 0.9	4.4 ± 0.9	8.1 ± 1.2	8.1 ± 1.2	8.1 ± 1.2
*Anthropometric measures*						
Bone stiffness index	79.3 ± 14.6	79.8 ± 15.5	79.5 ± 15.0	82.1 ± 12.5	80.5 ± 11.7	81.3 ± 12.1
Body height (cm)	116.0 ± 9.8	114.7 ± 9.4	115.4 ± 9.7	135.2 ± 9.0	134.7 ± 9.1	135.0 ± 9.0
Body weight (kg)	21.8 ± 5.4	21.1 ± 4.9	21.5 ± 5.2	32.5 ± 8.7	32.1 ± 8.3	32.3 ± 8.5
Fat-free mass (kg)	15.7 ± 3.6	13.9 ± 3.3	14.9 ± 3.6	23.1 ± 4.1	21.3 ± 4.0	22.2 ± 4.1
*Objectively measured PA and sedentary behaviours*						
Sedentary time (hours/day)	4.4 ± 1.5	4.5 ± 1.4	4.4 ± 1.5	5.6 ± 1.5	5.7 ± 1.5	5.7 ± 1.5
Light PA (hours/day)	6.4 ± 1.0	6.4 ± 1.0	6.4 ± 1.0	5.8 ± 1.1	5.8 ± 1.1	5.8 ± 1.1
Moderate PA (min/day)	38 ± 18	29 ± 14	34 ± 17	39 ± 19	29 ± 14	34 ± 17
Vigorous PA (min/day)	7 ± 7	6 ± 5	6 ± 6	9 ± 8	7 ± 6	8 ± 7
Moderate-to-vigorous PA (min/day)	45 ± 23	35 ± 18	40 ± 21	48 ± 25	36 ± 18	42 ± 23
PA levels (cpm)	653 ± 171	586 ± 147	622 ± 164	579 ± 169	507 ± 139	541 ± 158
Wearing time (average hours/day)	11.6 ± 1.7	11.4 ± 1.7	11.5 ± 1.7	12.2 ± 1.7	12.1 ± 1.7	12.2 ± 1.7
*Measures of muscle strength*						
Jumping distance (cm)				121 ± 25	114 ± 25	117 ± 25
Handgrip strength (kg)				14.4 ± 3.9	13.2 ± 3.5	13.8 ± 3.7
*Potential confounding lifestyle factors*						
Dairy products (frequency/week)	22 ± 12	21 ± 11	21 ± 12	20 ± 12	19 ± 12	20 ± 12
Daylight duration (hours/week)	11.0 ± 2.8	10.8 ± 2.6	10.9 ± 2.7	10.8 ± 2.6	10.7 ± 2.6	10.7 ± 2.6
*Reported PA and sedentary behaviours*						
* Continuous variables*						
Leisure time PA (hours/week)	17.8 ± 10.0	17.6 ± 9.8	17.7 ± 9.9	18.9 ± 9.8	17.2 ± 9.7	18.0 ± 9.8
Screen time (hours/week)	12.2 ± 7.0	10.6 ± 6.3	11.4 ± 6.7	15.2 ± 8.1	13.3 ± 7.2	14.2 ± 7.7
*Categorical variable*	*N* (%)	*N* (%)	*N* (%)	*N* (%)	*N* (%)	*N* (%)
High-to-moderate WBE	182 (22.6)	222 (31.4)	404 (26.7)	822 (58.3)	817 (52.9)	1639 (55.5)
No-impact WBE / no exercise	622 (77.4)	486 (68.6)	1108 (73.3)	587 (41.7)	727 (47.1)	1314 (44.5)

*cpm* average counts per minute, *N* number, *PA* physical activity, *WBE* weight-bearing exercises, *SD* standard deviation

Tables 2 and 3 present the results of models 1–4 for objectively and subjectively assessed variables, respectively. The explained variance of SI by PA, sedentary behaviour and muscle strength was about 18–19 % in preschool and 24–27 % in school children, which was consistent throughout the models. Regression coefficients were not or only slightly reduced by adjustment for FFM, MDP and daylight duration (model 2), PA, or respectively sedentary behaviour (model 3) and muscle strength (model 4).

**Table 2:** Multivariate linear regression investigating the association of accelerometer-based PA data with SI, by age group

	Model 1			Model 2			Model 3^a^			Model 4		
	Adjusted for age, sex, country			Model 1 + adjusted for FFM, MDP, daylight duration			Model 2 + adjusted for either PA or/and sedentary time			Model 3 + adjusted for muscle strength		
	β	*p* value	R^2^ (%)	β	*p* value	R^2^ (%)	β	*p* value	R^2^ (%)	β	*p* value	R^2^ (%)
Preschool children (*N* = 1512)												
Sedentary time (hour/day)	−0.84	0.010	17.9	−0.73	0.008	18.6	−0.37	0.28	19.1			
Light PA (hour/day)	0.11	0.77	17.5	−0.05	0.89	18.3	−0.47	0.26	18.6			
Moderate PA (per 10 min/day)	0.76	<.001	18.1	0.83	<.001	19.0	0.75	0.003	19.1			
Vigorous PA (per 10 min/day)	1.23	0.047	17.7	1.37	0.027	18.6	1.22	0.05	18.8			
MVPA (per 10 min/day)	0.59	0.001	18.1	0.65	<.001	19.0	0.58	0.003	19.0			
PA levels (per 100 cpm)	0.96	<.001	18.3	0.92	<.001	19.0						
FFM (kg)^b^	−0.41	<.001	17.9	−0.40	0.001	18.2	−0.40	<.001	19.0			
School children (*N* = 2953)												
Sedentary time (hour/day)	−0.60	<.001	24.1	−0.77	<.001	25.9	−0.42	0.015	27.0	−0.44	0.011	27.2
Light PA (hour/day)	0.26	0.17	23.8	0.43	0.025	25.5	0.004	0.98	25.9	0.09	0.69	26.3
Moderate PA (per 10 min/day)	0.81	<.001	24.8	0.94	<.001	26.7	0.83	<.001	26.8	0.78	<.001	27.1
Vigorous PA (per 10 min/day)	1.61	<.001	24.6	1.74	<.001	26.3	1.58	<.001	26.7	1.43	<.001	26.9
MVPA (per 10 min/day)	0.65	<.001	24.9	0.73	<.001	26.8	0.66	<.001	26.9	0.61	<.001	27.2
PA level (per 100 cpm)	0.84	<.001	24.7	1.02	<.001	26.7				0.96	<.001	27.0
FFM (kg)	0.44	<.001	25.3	0.45	<.001	25.3	0.50	<.001	26.7	0.41	<.001	27.0
Jumping distance (10 cm)	0.41	<.001	24.2	0.35	<.001	25.7	0.27	0.005	26.9			
Handgrip strength (kg)^b^	0.41	<.001	24.9	0.20	0.013	25.5	0.18	0.021	26.9			

*cpm* average counts per minute, *FFM* fat-free-mass, *MDP* milk and dairy products, *MVPA* moderate-to-vigorous physical activity, *PA* physical activity

^a^In Model 3, the *independent variables* were (in addition to Model 2) adjusted as follows: *sedentary time*: moderate and vigorous PA; *light PA, moderate PA, vigorous PA and MVPA:* sedentary time; *musculoskeletal fitness and FFM*: PA level; *PA level:* no additional adjustment for other accelerometer-based variables

^b^In Model 2, FFM was adjusted for MDP and daylight duration

**Table 3:** Multivariate linear regression investigating the association of reported PA data with SI, by age group

		Model 1			Model 2			Model 3^a^			Model 4		
		Adjusted for age, sex, country			Model 1 + adjusted for FFM, MDP, daylight duration			Model 2 + adjusted for either PA or SB			Model 3 + adjusted for muscle strength		
		β	*p* value	R^2^	β	*p* value	R^2^	β	*p* value	R^2^	β	*p* value	R^2^
Preschool children (*N* = 1512)													
Screen time (hours/week)		−0.07	0.22	17.4	−0.04	0.50	18.2	−0.04	0.50	18.4			
Leisure time PA (hours/week)		0.07	0.048	17.5	0.07	0.05	18.4	0.07	0.047	18.4			
WBE	Impact WBE	2.26	0.009	17.7	2.59	0.003	18.7	2.57	0.003	18.7			
Reference: no exercise/WBE													
School children (*N* = 2953)													
Screen time (hours/week)		0.002	0.92	23.7	−0.01	0.63	25.3	−0.01	0.54	25.5	−0.01	0.85	25.8
Leisure time PA (hours/week)		0.05	0.023	23.8	0.05	0.019	25.5	0.05	0.020	25.5	0.04	0.037	25.8
WBE	Impact WBE	1.73	<.001	24.2	1.67	<.001	25.7	1.66	<.001	25.7	1.49	<.001	26.0
Reference: no exercise/WBE													

*PA* physical activity, *WBE* weight-bearing exercise

^a^In Model 3, the *independent variables* were (in addition to Model 2) adjusted as follows: *screen time*: leisure time PA; *leisure time PA* and *WBE:* screen time

### PA behaviour and SI

We observed a positive association of accelerometer-based PA levels with SI in preschool (β = 0.92, *p* < .001) and in school children (β = 1.02, *p* < .001). When classified into PA intensities and based on model 3, our results suggest that a 10 min increase in MPA per day would lead to an about 0.8 unit higher SI in both age groups (*p* ≤ .001). With the same increase in VPA, the association with SI was about 1.6–2-fold as high as the association between MPA and SI in preschool (β = 1.22, *p* = 0.05*)* and school children (β = 1.58, *p < .001)*. LPA showed no clear association with SI in both age groups (see Table 2).

Considering reported PA, we observed higher SI values of about 2.6 units for preschool (*p* = 0.003) and 1.7 units for school children (*p* < .001) who participated in WBE compared to those performing no WBE or no exercise at all. In contrast, for leisure time PA, we observed a 0.07 (*p* = 0.047) and 0.05 unit (*p* = 0.020) increase in SI for an additional hour per week in preschool and school children, respectively (see Table 3).

### Sedentary behaviour and SI

Accelerometer-based SED was negatively associated with SI in preschool and school children. After adjusting for MVPA (Model 3), the association between SED and SI was reduced by about 50 % in preschool (β_Model2_ = −0.73, *p =* 0.008 versus β_Model3_ = −0.37, *p* = 0.28) and 45 % school children (β_Model2_ = −0.77, *p >* .001 versus β_Model3_ = −0.42, *p* = 0.015). No association was found between the reported screen time and SI.

### Muscle strength and SI

In preschool children, FFM was negatively associated with SI (β = −0.40, *p =* 0.001). In school children on the other hand, FFM and muscle strength were positively associated with SI. In the latter, an additional kg of FFM corresponded to a 0.5 unit (*p* < .001) higher SI. Furthermore, every 10 cm increase in jumping distance and every 1 kg increase in handgrip strength resulted in a 0.3 (*p* = 0.005) or 0.2 units (*p* = 0.021) higher SI, respectively.

## Discussion

This study provides quantitative evidence on the association of objectively measured PA and sedentary time as well as of muscle strength with SI based on a large sample of 2–10-year-old children.

Overall, especially high-impact PA such as WBE, which are mostly of high intensities, i.e. VPA, substantially contributed to a higher SI already from preschool age, while no such effect was found for LPA. The observed association of high-impact PA with SI in preschool and school children was comparable. Our data indicate a decline of the osteogenic effect of PA with decreasing intensity. While 10 min of additional VPA per day lead to an almost 2 % higher SI, the same increase in MPA showed a 1 % higher SI. In addition, our results indicate, that MVPA partially reduces the adverse effect of SED on SI.

Previous observational studies support our findings that time spent in VPA appears to be more strongly associated with indicators of bone strength in children than time spent in LPA, MPA or MVPA [9, 52]. Furthermore, our results show similar associations as found in studies that examined the impact of MVPA and VPA on bone mineral content (BMC) or density (BMD). For instance, the Southampton Women’s Survey, the only study found involving 4-year-old children, observed a 1.4 % higher BMC for 10 min of additional MVPA per day [5]. The Iowa study reported that, in 5-year-old children, an extra 10 min in VPA per day lead to a 3 % higher BMC. In 9–10-year-old children from the European Youth Heart Study, additional 10 min of VPA were associated with a 1–2 % higher BMD [9]. According to Kriemler et al., a change of 1.8–2.8 % in bone mass by PA or exercise intervention may be of relevance [8]. Following Kriemler et al., our results suggest that at least an additional 10 min in VPA or 20 min in MPA per day would be necessary to achieve a relevant increase in SI. However, it should be kept in mind that SI is more an indicator for bone strength than bone mass [34]. Nevertheless, on the one hand, such an increase in SI would be more realistic and relevant for children who have not reached their optimum SI for age and sex [28]. On the other hand, we hypothesize that the osteogenic effect in children who already perform high PA levels may reach a plateau after a certain time spent in these intensities. This would be an interesting issue to examine in a longitudinal perspective.

A stronger effect of MVPA or VPA on BMC in boys than in girls has been reported in previous studies [6–9]. For instance, comparing the highest tertile of VPA (72 min/day) to the lowest tertile (22 min/day), Kriemler et al. observed a 0.19 g difference of BMC in girls and a 1.22 g difference in boys. That is a 6-fold higher effect in boys than in girls [8]. Similarly, in the longitudinal perspective of the Iowa study, Janz et al. found that an additional 30 min of MVPA per day at age 5 lead to a 4.5 and 6.7 % higher BMC in girls and boys at age 8, respectively. That is an almost 50 % higher BMC accrual in boys compared to girls [6]. The researchers explained this sex-difference due to higher PA levels in boys [6, 8, 12]. Likewise, the boys in our study spent on average more time in MVPA than girls. Although we have found no moderating effect of sex on the association between PA and SI, we performed a sensitivity analysis stratified by sex, which was based on Model 3. The association of MVPA and VPA with SI was only slightly stronger in boys than in girls. For every additional 10 min in MVPA per day we observed an about 66 % higher increase SI in boys (β = 0.48, *p* < .001) compared to girls (β = 0.29, *p* = 0.06). For the same increase in VPA, boys had only a 19 % higher increase SI compared to girls (β_boys_ = 1.25, *p* < .001 vs. β_girls_ = 1.05, *p* = 0.025). On the one hand, a 66 % higher increase SI in boys is in line with the reported sex-difference from the Iowa study [6]. On the other hand, a 0.2 unit higher increase in SI for additional 10 min in MVPA, i.e. a 1.2 unit higher SI increase for an additional hour in MVPA appears to be a negligible sex-difference. Although our results do not indicate any sex-difference, previous studies suggested that boys might potentially have a higher genetically determined responsiveness of bones to PA and exercise compared to girls. However, until today there is no evidence of this in children [8].

Contrary to objectively measured PA, reported leisure time PA was only weakly associated with SI in our study. School children whose parents reported 4 h more in leisure time PA per week, i.e. approximately 30 min more leisure time PA per day, only showed a 0.3–0.4 % higher SI. The weaker association between reported PA and bone indices compared to objectively assessed PA is consistent with findings from the Iowa study [53]. However, we observed a strong positive association between subjectively assessed WBE and SI. Preschool and school children whose parents reported participation in WBE had a 3.2 and 2.0 % higher SI, respectively compared to children that did not participate in WBE or exercise at all. The beneficial impact of WBE on bone development already in preschool age was observed in a 12-month randomized intervention trial conducted in U.S. by Specker et al. (2004, *N* = 161). In the study, 3–5-year-old children who participated in a gross activity group (bone-loading and large muscle exercises) had greater increases in BMC and BA compared to those who participated in the fine motor activity group (non-bone loading activities) [54]. This observed osteogenic effect of objectively assessed WBE is in line with our results concerning parental-reported WBE. We conclude that both, objectively measured PA as well as reported WBE, appear to be valuable indicators to investigate dose–response relationships of the impact of PA on bone.

The osteogenic effect of high-impact PA such as WBE has been proven in many school-based intervention programs involving jumping exercises. A 3–8 % higher BMC was observed in children who participated in such programs lasting 7–9 months for at least three times per week compared to their peers who did not [13–15, 17]. Taking into account the reported periods of those school-based intervention programs, a dose–response effect of WBE on bone accrual can be suggested [2, 12–14, 18]. This suggestion is partly in line with the current WHO guidelines that recommend bone-strengthening exercises on at least three days a week [20]. However, there is a lack of evidence regarding the optimal dose of habitual PA for an adequate skeletal development in children. Our results indicate a positive association of MPA and especially of VPA with SI, although the prevalence of children who spent the recommended 60 min per day in MVPA was only about 20 % (see Table 4). In a case–control study, embedded in the IDEFICS project, we have observed that children who spent less than 30 min in MVPA per day, i.e. less than 4.2 % of their total PA, did have a 70 % increased risk for a low SI compared to children who spent more than 46 min per day, i.e. more than 6.7 % of their total PA in MVPA [55]. The latter result suggests that 30 min MVPA per day is not sufficient for an optimal SI. However, the optimal dose of habitual PA as well as of specific WBE programmes and their sustainable effect on bone accrual needs to be further investigated in longitudinal studies including intervention programs [15].

**Table 4:** Prevalence (%) of preschool (2- < 6 years) and school (6–10 years) children, who reached the World Health Organization (WHO) recommendation (2011) of 60 min/day moderate-to-vigorous physical activity (MVPA^a^, stratified by sex)

	Preschool children			Primary school children		
	Boys *N* = 804	Girls *N* = 708	All *N* = 1512	Boys *N* =1402	Girls *N* = 1535	All *N* = 2937
Prevalence (%) of children spending ≥ 60 min/day MVPA^a^	25.0	10.3	18.1	30.6	10.5	20.1

^a^MVPA based on the cut-off values proposed by Evenson (published by Trost et al., 2011) [42]

Our data support the beneficial osteogenic effect of muscle strength in school children. Currently, there are heterogeneous findings regarding the mediating role of muscle strength on the association between PA and SI [1, 8, 16]. After controlling for muscle strength, we only observed a slightly reduced association of MPA and VPA with SI. This may be due to the fact that jumping distance and handgrip strength may not measure muscle strength as accurately as other isokinetic methods (e.g. ISOMED 2000). However, the latter methods are used in the laboratory and are not feasible to measure muscle strength in children [56, 57]. Jumping distance and handgrip strength have been observed to be good proxies for muscle strength in the lower and upper limbs assessed in field studies [29]. Another explanation for the slightly reduced association of MPA and VPA with SI after controlling for muscle strength is due to the collinearity of PA and muscle strength. Since higher levels of PA should in general lead to stronger muscles it is difficult to disentangle the effect of muscular forces and the effect of physical impact caused by WBA on bone development.

Considering FFM as an indicator for skeletal muscle mass, our results confirm a positive association with SI only in school children [8]. In preschool age, FFM was negatively associated with SI. This may be explained by the contribution of the higher proportion of organ tissues and the reduced proportion of skeletal muscle mass to FFM in early life [30, 58]. Furthermore, we calculated FFM accounting for height, a variable that was recently reported to be negatively associated with SI in preschool age [28]. Thus, the negative association between SI and FFM we observed indicates that FFM is not a suitable indicator for skeletal muscle mass in preschool children.

The application of QUS in large-scale studies in children is scarce. The few studies that used QUS mostly applied different devices, assessed PA using questionnaires or examined adolescents [25, 59–61]. This limits comparability with our findings. Only the ChiBS study applied the same QUS device as the one used in our study and assessed PA intensities by accelerometry, using the same cut-offs, but set at 15 s epochs. In this study, a negative association of SED and a positive association of VPA with SI among 6–12-year-old children was observed [25]. These results support our findings.

While the strength of this study lies in the large sample size, the wide age range of children, and the application of parent-reported and objectively assessed PA, the interpretation of our data must consider the cross-sectional design.

We are aware of the limitation regarding the sub-optimal application of the accelerometer in our study. The minimum wearing time of three consecutive days for at least six hours per day may result in an underestimation of the true time a child spent in PA [5, 8, 53]. Nevertheless, the average wearing time per day was 11–12 h. Furthermore, the advantage of assessing VPA in children by using 15 s epochs has been reported previously [62]. Shorter epochs that vary below 15 s may better collect short and sporadic activity bouts that correspond to the natural activity pattern of a child [7, 23]. However, our sensitivity analyses revealed 43 and 16 % weaker associations between accelerometer-based PA and SI in preschool and school children, respectively, when using 15 s epochs compared to 60s epochs, taking the smaller sample size into account. Finally, due to the limited number of available accelerometers in the IDEFICS study, only 50 % of all children wore an accelerometer. However, preschool (*N* = 1731) and primary school children (*N* = 1791) who did not wear an accelerometer had comparable data on SI (preschool: 82.5 ± 16.3, primary school: 80.3 ± 12.5), reported leisure time PA (preschool: 17.3 ± 10.4 h/week, primary school: 18.8 ± 10.8 h/week) and screen time (preschool: 11.3 ± 6.7 h/week, primary school: 14.7 ± 7.5 h/week) compared to those who did (see Table 1).

The validity of the variable WBE is limited, because this information has been parental reported and does not include WBE during leisure time outside of a sports club. Thus, WBE may be underestimated. All the more remarkable is that we observed a strong association between WBE and SI in children.

Another limitation is the missing fitness data in preschool children that hindered us from comparing the impact of muscle strength on SI between preschool and school age.

Furthermore, the calculated variable daylight may not be the best proxy for the child’s exposure to sunlight, since a child may spend the entire time in doors and therefore not be exposed to sunlight.

Finally, we cannot be sure whether all children were pre-pubertal since no information on maturity stages was available. To rule out the positive effect of oestrogens on the bone’s sensitivity to PA in older girls, we performed sensitivity analysis in girls younger than 9 years and observed similar associations between PA and SI [18].

## Conclusion

Our findings suggest that the association between accelerometer-based PA and QUS-based bone indices may contribute towards determining potential dose–response relationships in children. This finding should be taken into consideration when planning further large-scale studies. Our study highlights the importance of high-impact and intense PA rather than light PA for optimizing SI, as a proxy for bone strength in 2–10-year-old children. The participation in WBE, or respectively 10–20 min of extra MPA and VPA per day appear to be sufficient for a relevant increase in SI.

## Abbreviations

ALPHA: Assessing Levels of Physical Activity
BMC: Bone mineral content
BMD: Bone mineral density
ChiBS: Children’s body composition and stress
cpm: Counts per minute
FFM: Fat-free mass
IDEFICS: Identification and prevention of dietary- and lifestyle- induced health effects in children and infants
LPA: Light physical activity
MDP: Milk and dairy products
MPA: Moderate physical activity
MVPA: Moderate-to-vigorous physical activity
PA: Physical activity
s: Seconds
SAS: Statistical analysis software
SB: Sedentary behaviour
SED: Sedentary time
SI: Bone stiffness
VPA: Vigorous physical activity
WBE: Weight-bearing exercises
WHO: World Health Organization
